# Mathematical modelling and discrete mathematics: opportunities for modern mathematics teaching

**DOI:** 10.1007/s11858-022-01339-5

**Published:** 2022-03-10

**Authors:** Gilbert Greefrath, Hans-Stefan Siller, Katrin Vorhölter, Gabriele Kaiser

**Affiliations:** 1 University of Münster, Münster, Germany; 2 University of Würzburg, Würzburg, Germany; 3 University of Hamburg, Hamburg, Germany; 4 Nord University Bodø, Bodø, Norway

**Keywords:** Graph theory, Modelling competences, Modelling days, Discrete model

## Abstract

Discrete mathematics and mathematical modelling, along with the educational discourse surrounding these, have many connections. However, ways that the educational discourse on discrete mathematics can benefit from the inclusion of examples of mathematical modelling and the accompanying discussion are currently under-researched. In this paper, we elaborate on the educational potential of examples of mathematical modelling based on the usage of methods from discrete mathematics, with a focus on secondary education. We first describe vertex-edge graphs as possible topics of discrete mathematics that are accessible at school level within modelling lessons. Secondly, in the context of a case study, we describe modelling activities with students at the end of lower-secondary education, using a classical problem of discrete mathematics originating from the Königsberg bridge problem. The students’ solution processes for this optimisation problem based on graph theory are described. Their approaches are examined referring to the phases of the modelling cycle, using the method of qualitative content analysis. We studied in particular the extent to which students use concepts related to vertex-edge graphs in specific sub-phases of the modelling process. The analysis allows the required sub-competences of modelling to be identified and the connection of these competences with discrete mathematics to be worked out. On the basis of this analysis, educational opportunities of teaching discrete mathematics and mathematical modelling are assessed. Overall, we point out the possibilities and opportunities for using examples from the field of discrete mathematics to acquire modelling competences and to foster the linkage of mathematical modelling and discrete mathematics at school level.

## Introduction

Making connections between discrete mathematics and mathematical modelling holds great potential from the perspective of mathematics education, and this potential is further elaborated in the context of this paper. Using discrete mathematics methods allows for a broader—and in part less formal—approach to working with mathematical models, since, for example, the concept of infinity can initially be avoided. Limits and infinity usually create high learning barriers and epistemological problems for secondary school students or for students at university (Goldin, 2004). Methods of discrete mathematics in connection with competence in mathematical modelling should be an essential part of a modern curriculum (Hart & Martin, 2018), as discrete mathematics is considered to have high value in mathematical education (Beutelspacher & Zschiegner, 2014).

We first present different approaches to discrete mathematics in connection with mathematical modelling in secondary education and explain the potential for solving concrete modelling problems with discrete models. We then detail the educational aspects of discrete mathematics and mathematical modelling. These are manifested at levels of content, progressive technological development, and discussion of discrete models in the context of modelling processes. This analysis leads to the question of the role of discrete ideas concerning students’ modelling processes. In a case study, we investigated the actual use of these ideas in detail. In this way, the necessary sub-competences of modelling could be identified and connections with discrete mathematics using the example of a graph-theoretical problem could be worked out, to show opportunities and possibilities for promoting the link between mathematical modelling and discrete mathematics in school, thereby potentially strengthening the teaching and learning of both discrete mathematics and mathematical modeling.

## Mathematical modelling and discrete mathematics in educational discussions

There is a long tradition of requiring real-life applications to be included in mathematics education, and mathematical modelling and related modelling competences have finally become a central component of national curricula (Kaiser, 2020; Vorhölter, Greefrath, et al., 2019).

In the past decades, there have also been repeated calls for the consideration of modern mathematical subject areas for mathematics education that are also attributed proximity to mathematical modelling. In addition to stochastics (Chick & Watson, 2003), these themes include discrete mathematics in particular (Anderson et al., 2004; Dolgos, 1990).

Even during the 1980s and 1990s, there was intensive discussion about discrete models in the International Community of Teachers of Mathematical Modelling and Applications (ICTMA) (e.g. James & Wilson, 1986; Street & Street, 1998; van den Heuvel & Krabbendam, 1991; Ziegenbalg, 1984). In these contributions, teaching proposals with discrete models of growth behaviour, graph theory and difference equations were presented from a modelling perspective. In the educational discussion over the following decades, empirical studies were carried out that integrated mathematical modelling and discrete mathematics, and researchers pointed out that discrete mathematics would be indispensable for a modern curriculum (Hart & Martin, 2018).

In the following sections, we work out the connection between discrete models and mathematical modelling.

### The current discourse on mathematical modelling

**Fig. 1:**
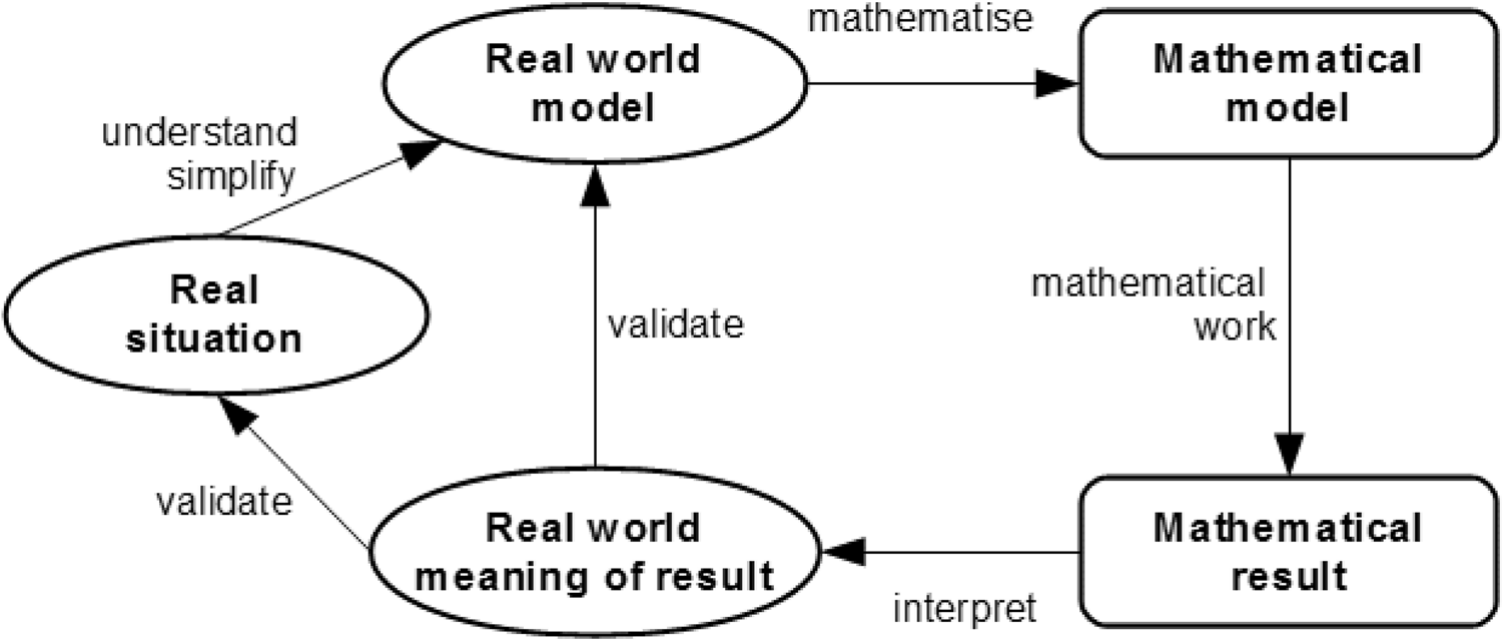
Modelling cycle, from Kaiser and Stender (2013)

According to Pollak (1977, p. 255 ff.), mathematical modelling refers to a specific aspect of applied mathematics that is currently described using repeated work through a modelling cycle. The modelling cycle describes the expected activities of students and provides metacognitive help (Stillman, 2011). Currently, there exist many descriptions of the expected modelling activities by students. One detailed modelling cycle was developed by Kaiser and Stender (2013), whose application is described as an example in a case study in the second part of the paper. However, it should be noted that there exist many nuances of the modelling cycle that we cannot unpack due to space limitations (for a detailed overview, see, e.g., Kaiser, 2017). In this modelling cycle, the process of the model’s individual development from an initial real-world situation to the actual model is presented as the first phase, which usually includes several simplification steps. This modelling phase is followed by translation into mathematics, from which a mathematical model is developed. With the help of this model, a mathematical solution is then determined, and this finally has to be related to the real problem again by interpreting and validating a solution in the original real-world situation (see Fig. 1). This modelling cycle describes the various sub-processes of mathematical modelling, such as understanding and simplifying, in more detail than modelling cycles from applied mathematics (e.g., Ortlieb, 2004; Pohjolainen & Heiliö, 2016).

An important construct within the modelling discourse is the competence of students across various educational levels in modelling. This competence enables them to identify a problem in a real-world situation, translate it into mathematics and interpret and validate a solution in relation to the given situation (Niss et al., 2007, p. 12). Modelling competence also refers to the willingness to work on mathematical modelling problems through mathematical means, and it is therefore distinguished from ability (Kaiser, 2007; Maaß, 2006).

The construct of modelling competence is currently characterised by sub-competences that are connected to the various phases of the modelling cycle (cf. Fig. 1). In addition, more comprehensive aspects are also included in the construct, such as the ability to carry out a complete modelling process independently, use metacognitive knowledge and structures and critically reflect the modelling cycle (for an overview on this discourse, see Cevikbas et al., 2021).

As a result of empirical studies, many researchers have pointed out that modelling tasks present difficult and complex work for students (see Kaiser, 2017, for an overview), and there are many barriers that students have to overcome (Galbraith & Stillman, 2006). Using metacognitive strategies can help students remove cognitive barriers during the modelling process. They can focus on the selection of a strategy, including the incorporation of alternative strategies. Eventually, the successful implementation of the chosen strategy will depend on both the students’ individual resources (in terms of strategies) and the task (Stillman, 2011; Vorhölter, Greefrath, et al., 2019; Vorhölter, Krüger, et al., 2019).

### Discrete mathematics in school

Discrete mathematics in school deals with configurations that can be described by a finite or countable set of relations. For example, the fields of combinatorics, number theory, graph theory, game theory, cryptography and statistics may all be counted as discrete mathematics (Ouvrier-Buffet, 2020). Currently, the advantages of discrete mathematics for mathematics teaching are seen on three levels, namely, the “content, process, and affect goals of mathematics education” (Hart & Martin, 2018, p. 18). The first level consists of the content, which is also evident in the analysis of new, interesting, and relevant contexts. Here, one can consider various important problem types, such as from the areas of combinatorics, iteration and recursion, as well as graph theory.

The second level refers to process-related competences such as reasoning, communicating, problem-solving and modelling, and it can be promoted particularly well through discrete mathematics (Hart & Martin, 2018). These processes form a central component of current educational standards in various countries (KMK, 2012; NCTM, 2000). At this level, discrete mathematics content can also be considered a tool for mathematical work from a process-related perspective. For example, vertex-edge graphs can be considered as modelling tools (Greubel et al., 2020; Thomas et al., 2015), and it would be expected that mathematical modelling with discrete models would be a central topic area in school and in the modelling discussion. However, only a few empirical studies on this topic have been carried out so far.

The third level refers to affect-related goals. Discrete mathematics can help students “see mathematics in a new light” (Hart & Martin, 2018, p. 17), and it is thus seen as particularly suitable for offering teachers a new image of mathematics. In this way, it enables them to motivate their students about the subject (DeBellis & Rosenstein, 2004).

Many suggestions for topics that are suitable for teaching secondary mathematics have been developed over recent decades. The possibility of using challenging problems that are at the same time easy to understand is emphasised (Anderson et al., 2004). In addition to many inner-mathematical problems from number theory, suggestions from coding theory have also been developed, such as on the EAN and ISBN systems. For secondary school, and partly also for primary school, graph theory in particular is highlighted as a possible subject area, as it is easily accessible for students at nearly all grades, and represents new content outside the curriculum that can be worked on without prerequisites (Gibson, 2012). By using graphical representations, graph theory may be as intuitively accessible as certain algebraic problems (Steele, 2008). Students are often able intuitively to suggest mathematical generalisations (Amit & Neria, 2008). Even more mathematically complex concepts such as Euler paths—i.e., edge sequences of a vertex-edge graph in which each edge is traversed exactly once without the start and end nodes having to be identical—can be easily taught with the help of illustrative problems. Vertex-edge graphs are therefore currently introduced to students as young as 12 years old (van den Heuvel & Krabbendam, 1991). Successful examples described for mathematics teaching use a variety of mathematical ideas from, among others, elementary graph theory, including complete vertex-edge graphs and Euler paths (Street & Street, 1998). In universities, the results of an empirical study suggested that the inclusion of graph theory in mathematics teaching would be beneficial for the development of students’ modelling skills (Medová et al., 2019). For teaching mathematics at school, graph algorithms in particular are suggested, also with the inclusion of visualisation through technology. For example, the questions of how optimally to drive a rubbish collection car (Geschke et al., 2005) or how to evacuate a building (Ruzika et al., 2017) are addressed with spreadsheet analysis and dynamic geometry. The examples also show another potential of discrete mathematics and mathematical modelling in dealing with optimization problems that have traditionally been solved with functional descriptions and calculus. Optimization problems are easily accessible and of high practical relevance, and have motivational power for students. There is a large number of examples of combinatorial optimization problems for students in discrete mathematics (DeBellis & Rosenstein, 2004; Schuster, 2004). The advantages of this topic are also seen especially at the second and third levels, that is in the process-oriented acquisition of competences and affect-related goals. In this context, the promotion of students’ motivation is emphasised and the connection of mathematics with real life is considered important (Ferrarello & Mammana, 2018).

### Connecting educational aspects

Connecting aspects of the different approaches of mathematical modelling and discrete mathematics for mathematics education can be identified from different perspectives.

A connecting perspective is of a *content-related nature*: “The power of discrete mathematics lies in mathematical modelling” (Hart & Martin, 2018, p. 5). This connection has also been recognised as relevant for mathematics education. In the 1980s and 1990s, teaching proposals for both mathematical modelling and discrete mathematics were discussed more intensively, and discrete models were seen as having particular potential for modelling (James & Wilson, 1986; Street & Street, 1998).

*The advancing technological development* is another perspective for considering discrete mathematics in the modelling discussion. Understanding how computers and their applications work requires knowledge of discrete mathematics in the context of modelling (Pollak, 2007). In the context of teaching discrete mathematics, the singular possibilities offered by technology have been pointed out (Durcheva & Varbanova, 2017; Weigand, 2004, 2014). This is also the case in the context of mathematical modelling (e.g., Greefrath et al., 2018; Keune & Henning, 2003; Sinclair & Jackiw, 2010). In this regard, some empirical results have already been reported (Hankeln & Greefrath, 2021). For example, in a case study, Greefrath and Siller (2017) observed students working on a reality-based task with GeoGebra. They studied the phases of the modelling cycle in which digital tools were used, and the activities that were carried out using these tools, during modelling activities. They found out that the use of digital tools took place mainly within the phases of mathematising and mathematical work. In the context of a short experiment to investigate the potential of introducing complex dynamical systems into curricula, it was shown that the transition to working with a computer simulation system would require one or more types of processes of discretisation, for example, referring to time or space (Caron, 2019). Already as early as in the 1980s, Ziegenbalg (1984) emphasised the usage of discrete models in the context of computer simulation in mathematics education.

A third perspective is the discussion of the *choice of discrete models in the context of modelling processes*. For example, James and Wilson (1986) and Ziegenbalg (1984) noted a greater proximity to real-world problems when modelling with discrete models compared to continuous ones. Nevertheless, James and Wilson (1986) emphasised that students should not be restricted to discrete or continuous models, and that a discrete model should not be considered a rough numerical approximation of a continuous model.

Recent empirical studies have encountered more differentiated results. For example, based on an evaluation of student difficulties, Castillo-Garsow et al. (2013) noted that continuous ideas of change might be more powerful than discrete ones. However, this need not be a contradiction, as discrete mathematics can also be used to specifically model continuous structures and thus provide a new way of looking at specific phenomena (Ouvrier-Buffet, 2020). Niss (2013) described discrete models as helpful in the course of working on data-based modelling problems—especially at the beginning of the mathematisation phase—that could then also be further developed into continuous models later on.

In a study to support the understanding among grade 10 students of rate of change and velocity using discrete representations with scatter plots, qualitative analyses showed that by independently carrying out modelling processes, the basic principles of infinitesimal calculus could be developed from the students’ reasoning about motion when supported by discrete representations (Doorman & Gravemeijer, 2009).

Observations in modelling problems on population growth by groups of students also showed a differentiated picture of the benefit of discrete models. While discrete models, which are rarely taught in school, would also be possible for population modelling, the students did not consider this option at the beginning and instead formulated a known function from their mathematics lessons. Another group, however, used a discrete model with a recursive formula, instead of a continuous model that they could not handle, and succeeded with the discrete approach (Kaiser et al., 2011). Overall, the actual use of discrete models probably depends on the mathematical methods available.

To summarise the current state of the art about the usage of discrete models within modelling activities, it can be noted that there are only a few empirical studies available, and these are based on smaller case studies. There exist empirical studies with students from secondary school, for example, on combinatorial problems such as the question of how many ways three flavours can be selected from six different options. Although there was a reference to reality, modelling was not at the core of this study (Coenen et al., 2018).

Therefore, there is a strong need for empirical research into the actual use of discrete approaches within modelling processes, evaluating the potential and pitfalls of the usage of discrete mathematics in mathematical modelling activities in schools.

## Research questions of the case study

While, on the one hand, the connecting elements of discrete mathematics and mathematical modelling are emphasised at the level of teaching proposals and in the field of technology, on the other, recent empirical studies show differentiated results while using discrete models in mathematical modelling processes (Castillo-Garsow et al., 2013; Kaiser et al., 2011). Graph theory is seen as a particularly appropriate area for studies of authentic discrete modelling problems, since it is a central area of discrete mathematics that is easily accessible and can be treated without prerequisites, and there is usually no prior experience of it in school (cf. Sect. 2.2). In the empirical study described in the following sections, we examine in depth students’ concepts when working on discrete modelling problems from the area of graph theory. The aim of the study was to investigate which phases of the modelling cycle require particular attention (cf. Niss, 2013). Specifically, the following research question was examined:

*To what extent do students use concepts related to vertex-edge graphs in specific sub-phases of the modelling process?*

## Method

### Design of the study

**Fig. 2:**
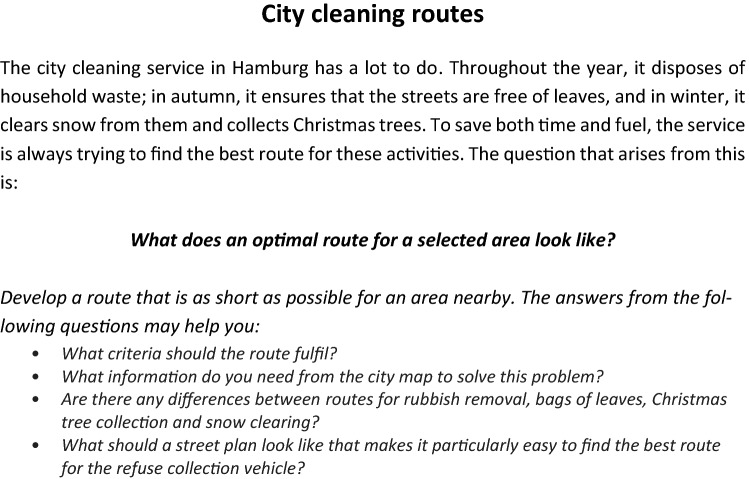
City cleaning task

The study was conducted in a higher-track school in Hamburg (Germany) during so-called modelling days. These modelling days have been held for many years by the Mathematics Education Working Group at the University of Hamburg. They last for 2 days, directly after the winter term in February. Every year, entire 9th grade classes from different schools take part. For this purpose, pre-service teachers were trained at an educational seminar that focused on teaching modelling (Vorhölter, Greefrath et al., 2019). During the modelling days, the students were given a task and were observed while working on it in groups. The study we describe in the following took place during a period when schools were closed due to the coronavirus pandemic; therefore, the students collaborated digitally using a video conferencing tool. They worked on a complex modelling problem that they had previously selected from three that were provided. The task consisted of a problem related to ‘city cleaning’ (see Fig. 2).

The problem was provided both on paper and as a video. During their work, the students were videotaped and supervised by two pre-service mathematics teachers who were asked to use the principal of minimal help. The working phase ended with the preparation of a presentation of the results of their work.

As the aim of the task was to support independent modelling activities by the students, no further guidelines were given to them. This meant that they could use all aids, especially information sources, but did not have to do so.

The problem was chosen because it is a reality-based modelling problem that can be solved with ideas from graph-theory by grade-9 students without any prior knowledge. Moreover, central graph-theoretical concepts such as Euler paths can be used to find a solution (see Sect. 2.2.).

**Fig. 3:**
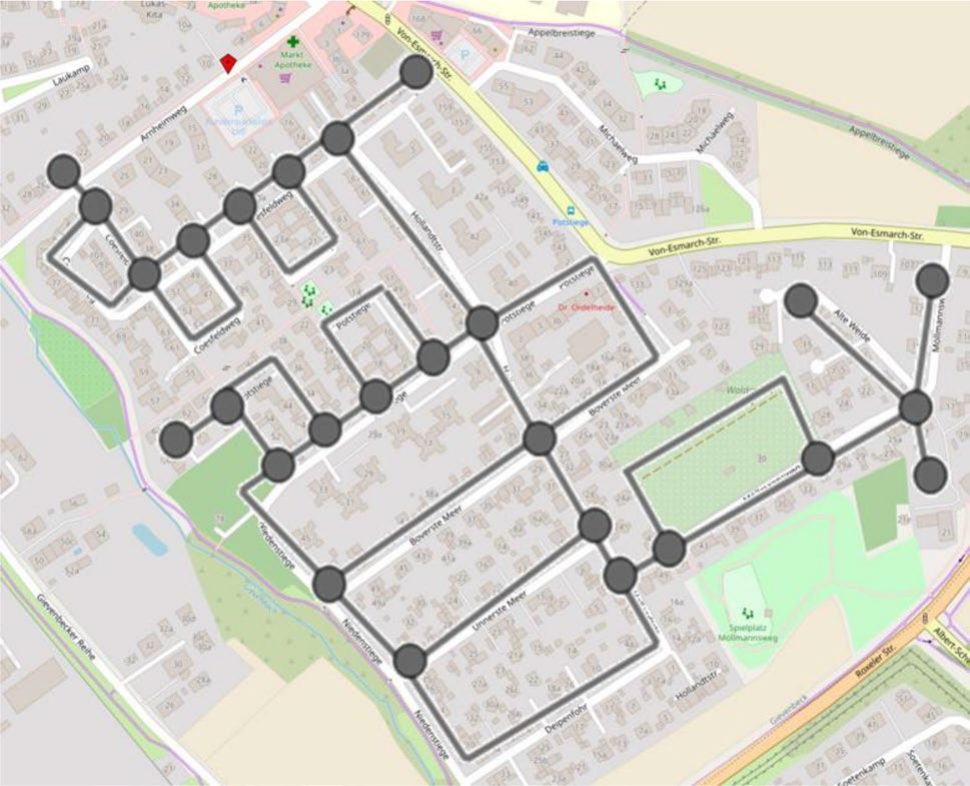
Map with vertex-edge graph of the road network (Map by Openstreetmap, licence CC-BY-SA 2.0)

**Fig. 4:**
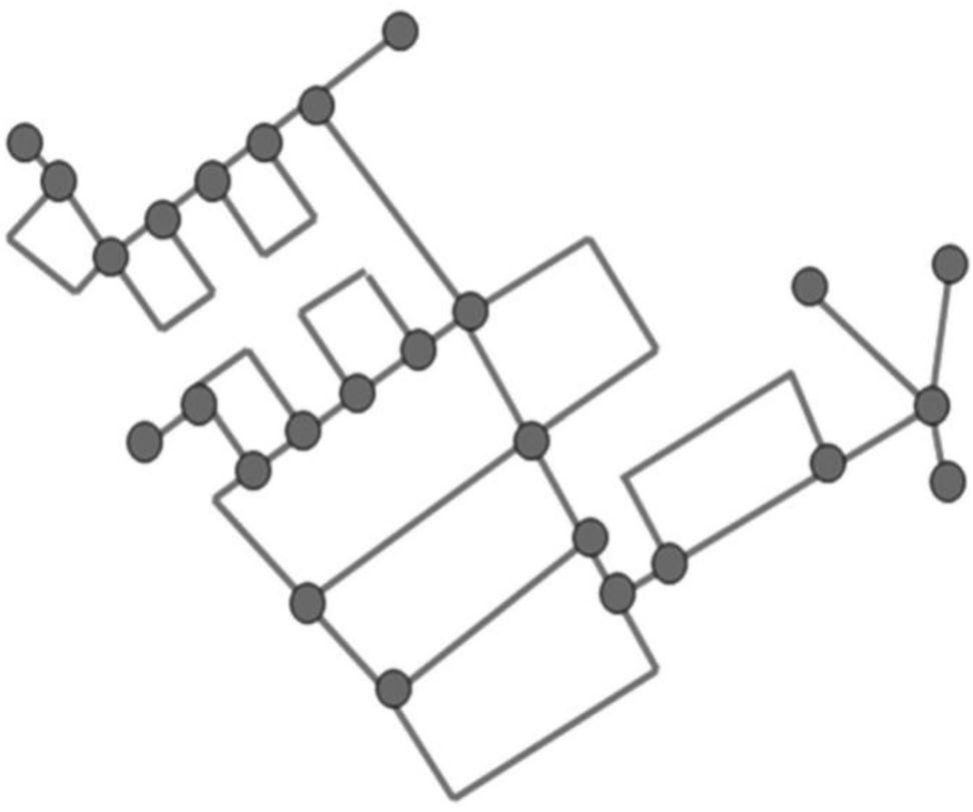
Vertex-edge graph of the road network

**Fig. 5:**
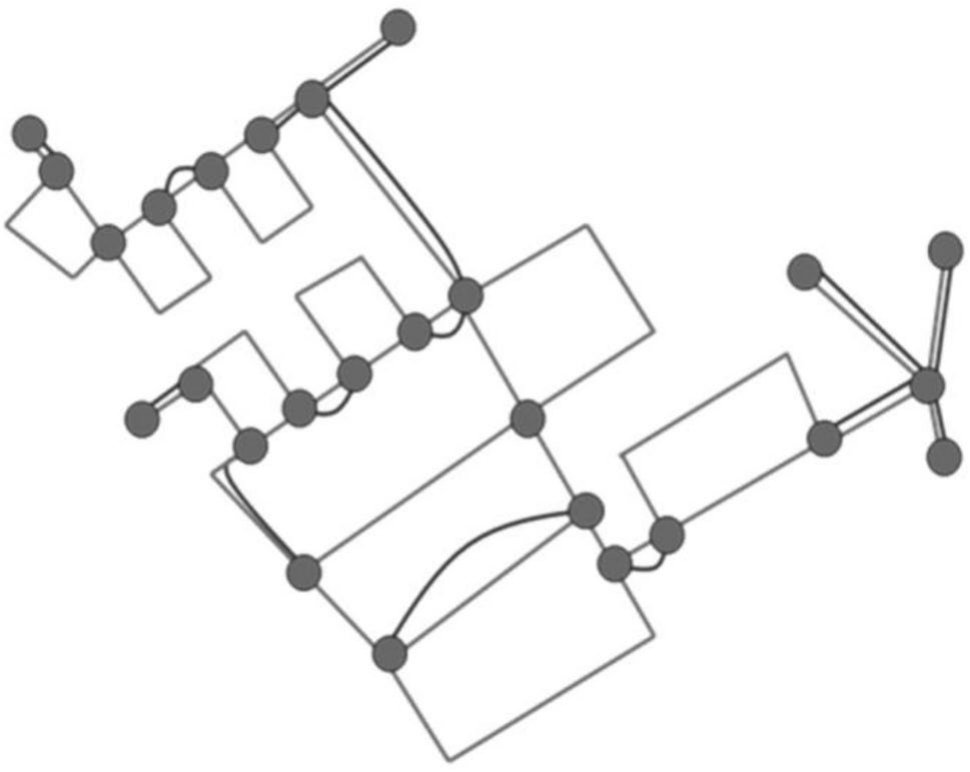
Vertex-edge graph of the road network with additional edges (Euler path)

A vertex-edge graph seems to be especially appropriate to support problem-solving because of its visual nature. After selecting the area of the city map to be cleaned, the students could show the road network in a simplified form as a vertex-edge graph, allowing them to describe the streets as edges and the intersections as nodes (see Fig. 3). A further simplification of the problem can now be achieved by focusing only on the vertex-edge graph (see Fig. 4). In further work, the degree of the nodes can be used directly to decide at which points roads must be covered twice. A possible vertex-edge graph with matching additional edges is shown in Fig. 5. For this purpose, a variant can then be determined in which the sum of these edges is as small as possible. With the help of this vertex-edge graph, a suitable Euler path can be found. However, it was not expected that the students would reach this point in their modelling activities.

### Sample and data collection

All students who had worked on the city cleaning task were asked to participate in the study, and all the reports of the groups in which all students volunteered to take part, were taken into account. Thus, the data base consists of video recordings of 8 female students of a grade-9 class (aged 14–15 years) in a higher-track secondary school. The students worked together in two small groups of four. The work phase comprised 9 h spread over two school days. Group 1 worked on the task for a total of 6 h, 57 min; Group 2 for 8 h and 37 min.

The four girls in the first group chose as their cleaning ground an area around the home of one group member, which was also very familiar to two of the others. They argued strongly based on real facts (such as parked cars, number of trees, traffic lights) and abstracted only slightly from these real facts. They regularly made sure that they were considering the task correctly, and referred in particular to the questions at the end of the task. They achieved their results by measuring different distances with a ruler.

The second group chose a sub-area that was prominently located in the city centre, although its members were not very familiar with it. They intensively discussed the start and end points, but their arguments were based on experiments, not on theoretical considerations. Parallel to the discussion of optimal start and end points, they discussed the weighting of the edges. In the first step, they considered the length of the route; in the second step, the time needed to drive through the route (caused, for example, by traffic lights and road works) seemed relevant. After attempting the task once, they did not go back to it.

### Evaluation method

Qualitative text analysis based on the approach by Kuckartz (2014) was used to evaluate the video data. Through a theory-guided and methodologically prescribed procedure, existing theoretical prior knowledge can be used. At the same time, the possibility of considering the empirical material in the evaluation process provides the necessary openness for considering new categories. The relevant scenes of the videos were transcribed according to a content semantic transcription system based on the approach developed by Dresing and Pehl (2018, p. 20 ff.) and coded by two experts from the field of mathematics and two from the field of mathematics education consensually in a deductive–inductive coding process. For this purpose, sampling units comprising sentences or whole sections of text on a certain topic were generated and then systematised in terms of content. Through this systematisation, categories with an abstract, classifying character and which reflected the content of the respective text passages were identified.

For this purpose, the phases of the modelling process in the video were first coded based on deductive codes. Analogous to a modelling activity diagram developed by Bergman Ärlebäck and Bergsten (2010), the course of the modelling process could be analysed focusing on specific sub-phases. Likewise, scenes in which concepts related to vertex-edge graphs (see Sect. 4.3.2) were reconstructed and presented. Thus, two codes were assigned to each scene relevant to the research question, namely, one related to the modelling process, the other to concepts related to vertex-edge graphs.

#### Category system for modelling processes

For the development of the category system for coding the sub-phases in the modelling process, the frequently used modelling cycle of Kaiser and Stender (2013), and the phases described there, were used. The categories were as follows: understanding, simplifying, mathematising, working mathematically, interpreting, validating and communicating. In the following, we have listed detailed descriptions in order to clarify the nature of these sub-competences (Greefrath et al., 2013, p. 19; Kaiser, 2007; Maaß, 2006):

- Understanding: Students construct their own mental model of a given problem situation and thus understand the question.
- Simplifying: Students make assumptions related to the situation, identify influencing variables, establish relationships between the variables, and search for relevant information.
- Mathematising: Students transfer the relevant quantities and relationships—simplified, if necessary—into a mathematical model and choose a suitable mathematical form of representation for this.
- Working mathematically: Students apply heuristic strategies and mathematical knowledge to solve the mathematical problem.
- Interpreting: Students translate mathematical results into extra-mathematical situations, generalise solutions developed for specific situations and represent problem solutions appropriately in language.
- Validating: Students check and reflect on solutions found, revise parts of the model if solutions to the situations are not appropriate and consider if other solutions or models are possible.
- Communicating: Students relate the answers they find to the real-world situation, and thus answer the question.

#### Category system for concepts related to vertex-edge graphs

For the discrete approaches, open codes were first identified and then further developed into categories (see Table 1).

**Table 1:** Categories of concepts related to vertex-edge graphs

Concepts related to vertex-edge graphs	Description
Edges	This category includes all codes in which students look more closely at the edges of the vertex-edge graph (not the nodes)
Metrics	This code is assigned to the weighting of road sections. This may be the length of a road or the presence of traffic lights or roadworks, etc., for which these roads should be avoided if possible (or at least should not be covered twice)
Effectiveness: Duplications	Consideration of roads covered twice
Effectiveness: Omissions	Consideration of omitted roads
Cover	Proportion of road coverage by the vertex-edge graph
Vertex-edge graph structure	This category includes all codes in which students look more closely at the structure of the vertex-edge graph
Subgraph	Considerations for viewing a sub-graph
Graph complexity	Development of basic insights into which a Euler graph might be created become apparent
Duplicates	Considerations of road sections covered twice, whereby the double driving is explicitly not done for reasons of time or path saving (for example, because in reality it seems easier to turn right instead of turning left)
Start and end points	Considerations of where the start and end points should be; also considerations of tour or circle
Directed graph	Concrete: Consideration of one-way street
Passage sense	Consideration of whether the graph should be covered from A to B or from B to A (e.g. because of left turns)
Generalisation	Discussion of transferring the model to other city districts or to other real situations

## Results

The students took different paths while working on the task and also achieved different results.

**Fig. 6:**
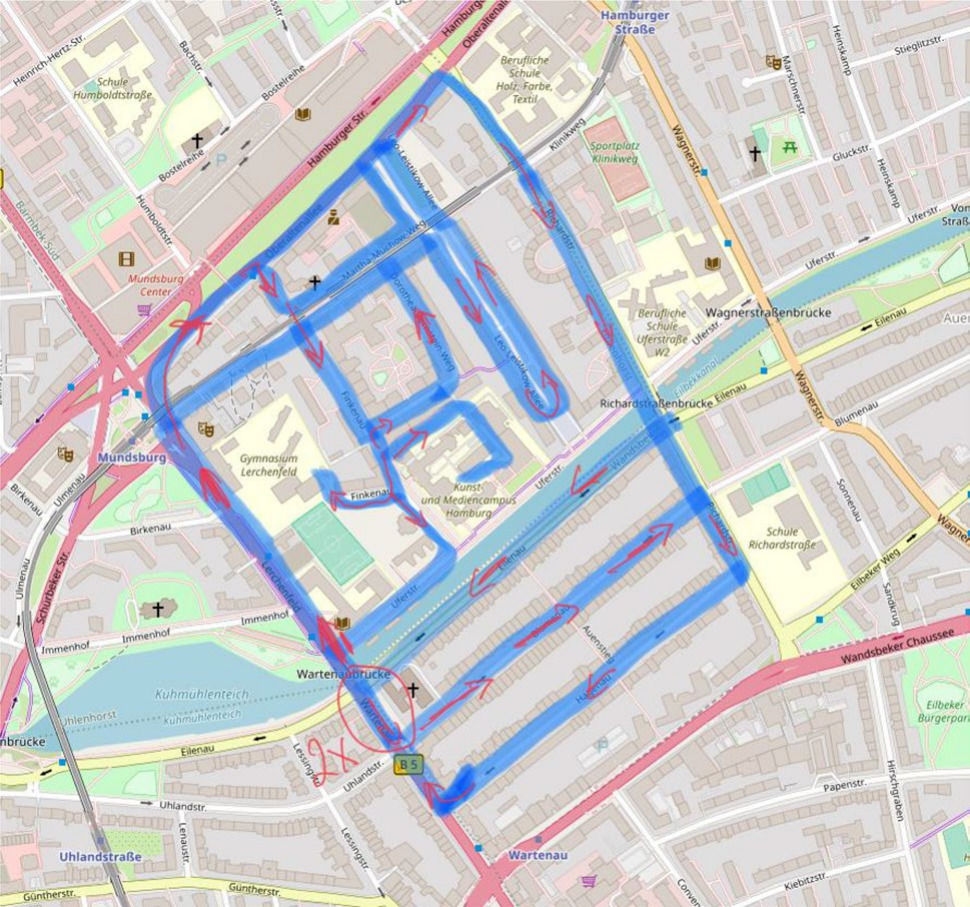
Road map with edge markings (Map by Openstreetmap, licence CC-BY-SA 2.0)

They approached the problem intuitively and worked with the concrete city map, from which they chose a specific section. While Group 1 chose an area around their homes, Group 2 chose one in the city centre. They marked in blue the streets that had to be passed through. They then selected a starting point and marked the possible routes with red arrows. In principle, they worked with vertex-edge graphs, but did not indicate the special role of the corners. Here, considerations were made as to which roads had to be covered twice and how these distances could be kept as short as possible. Such a sketch is shown in Fig. 6.

The use of concepts in the context of vertex-edge graphs can be divided into two central groups referring to edges and graph structure. Overall, the ‘effectiveness’ (see Table 1) of focusing on edges was considered very frequently, for example, when students discussed the question of not covering the longest distance twice.

In the following transcript excerpt, translated by the authors, the students talked about the question of whether driving down a road twice would make sense (i.e., driving through the edge twice):

## (Group 1, Part 1, Positions 49-52)

S2*: So you just drive this little piece twice. Is the question ... does that make sense?

S1*: Not really.

##

This section was coded as ‘Consideration of roads covered twice in the context of edges’ (Codes: Effectiveness: Duplications). The students were in the phase of mathematising with regard to the modelling processes.

Elsewhere, the students discussed the question of whether one should not run the longest distance twice:

## (Group 2, Part 2, Items 36-37)

S2*: I just took this one because it’s the longest route that you don’t have to take twice. But you couldn’t start here and end there either, because you don’t have to travel the final distance twice.

##

This section was also added to the codes ‘Effectiveness: Duplications’. However, the students were already in the mathematical work phase here because parallel calculations were being carried out. Such passages were identified 11 times in Group 2.

Another result was the frequent usage of metrics by the students. Here, the weighting of road sections was considered: it was discussed how fast one could cover these roads. Different aspects of reality that had an influence on the weighting of the roads (edges) were considered.

However, it should be noted that the usage of metrics occurred significantly more frequently in Group 2 than in Group 1. The third concept that was used frequently—especially in Group 2—was one of start and end points. In the transcript, it is discussed where these should be. An example comes from Group 1:

## (Group 1, Part 1, Items 42-48).

S1*: And why are we starting there?

S3*: Because ... because you can only start from there.

S2*: Because it’s a good place to start, because of all the one-way streets.

##

The students were talking about considerations of where the start and end points should be placed, in relation to the overall structure of the vertex-edge graph. At the same time, mathematisation activities took place with the aim of developing a mathematical model.

In addition to the consideration of the concepts concerning edges, and the structure of the vertex-edge graph, there was also discussion in a few cases about the transfer of the model to other situations (only in Group 1). Usage of the concept of nodes could not be identified.

**Fig. 7:**
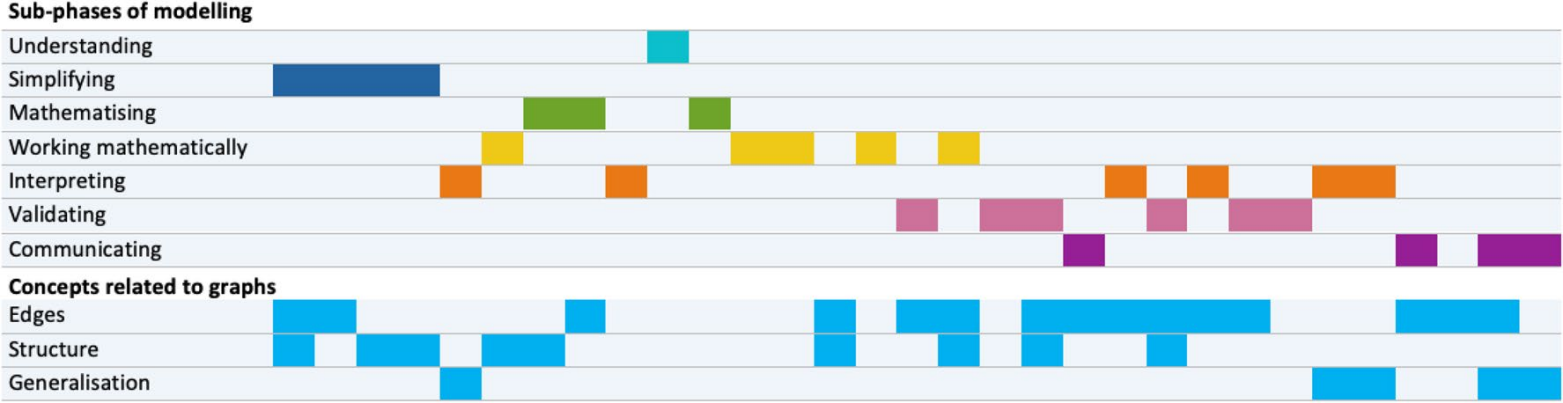
Work of group 1 over time

**Fig. 8:**
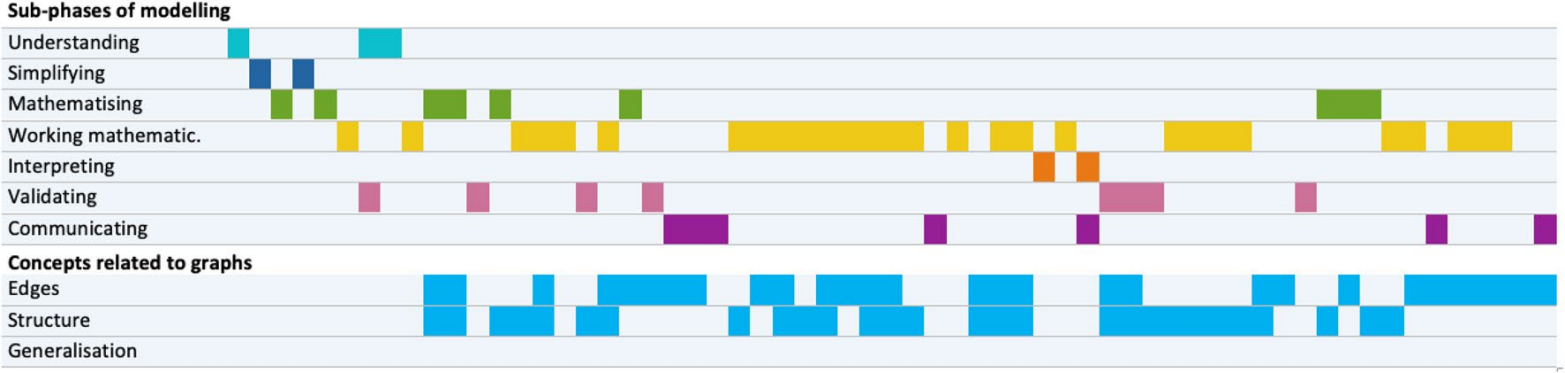
Work of group 2 over time

In both groups, all 7 sub-competences of mathematical modelling could be identified. In Figs. 7 and 8, and similarly to the ‘modelling activity diagram’ of Bergman Ärlebäck and Bergsten (2010), the first seven lines show the sub-phases of modelling over time. It can be seen that the seven phases of modelling are not carried out in the given order. In the three lines below, the three main categories of concepts related to vertex-edge graphs are also shown over time. This makes it clear which concepts related to vertex-edge graphs could be identified at different phases in the modelling process. The two extracts from the work of Group 1 shown above are from the first mathematisation phase (shown in green) in Fig. 7 and refer to the edges in the first case and to the structure of the graph in the second case. Overall, there is only one mathematisation phase by Group 1, in which concepts on graphs also could be identified at the same time.

Group 2 changed more frequently between the sub-phases of the modelling cycle than Group 1. While for Group 1, no focus on the modelling sub-phases could be identified, Group 2 showed a focus on working mathematically. Both groups also discussed concepts related to vertex-edge graphs over a long period of time. It should be noted that in Group 1, interpreting and validating in connection with concepts on graphs over a longer period of time also played a role, whereas in Group 2, these activities were used only rarely. In Group 2, no generalisation could be identified for concepts related to vertex-edge graphs.

The simultaneous coding of the modelling sub-phases, in which the students worked intensively and there was usage of concepts with reference to vertex-edge graphs, enabled a detailed analysis regarding in which sub-phases of the modelling cycle certain concepts were discussed. In the process, different emphases emerged for the two groups studied. In the following, both groups are presented individually.

In Group 1, there was no reference to vertex-edge graphs recognisable—i.e., comprehension of the problem—during the typical first phase of modelling (see Table 1). These references emerged during the simplification phase. Here, different aspects were used (sense of passage, start and end point, subgraph) that were not explicitly used later; nevertheless, the group obviously found its way to a solution. Mathematising activities were carried out with the use of four different concepts related to vertex-edge graphs. The effectiveness argument was used across all decisions in the subsequent sub-phases from the mathematisation phase onwards. In particular, duplications and omissions of roads (edges) were discussed here. Within the validation activities, different concepts related to vertex-edge graphs were discussed intensively in this group. An important aspect besides effectiveness was the complexity of the vertex-edge graph, which was identified in four different sub-phases of the modelling process. In the following transcript excerpt from Group 1, the students discuss the basic question of what a road network that is easy to navigate would look like. It relates to the complexity of the vertex-edge graph as well as to the simplification phase.

## (Group 1, Part 1, Items 12-14)

**Fig. 9:**
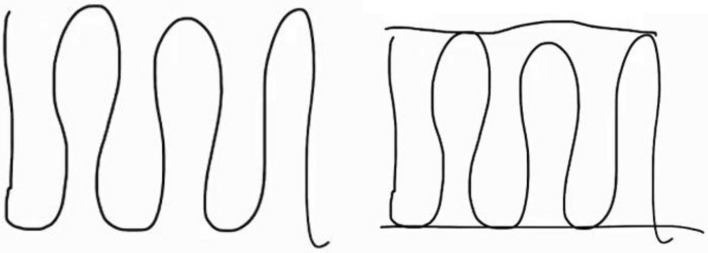
Student’s drawing

S2*: So if a road network ... [S2 draws, see Figure 9] ... the black is now a road network. If that were the case, it would be the easiest, wouldn’t it? ... But that doesn’t really exist; there’s usually also something like that [drawn] here.

##

To summarise, a detailed overview of concepts related to vertex-edge graphs in the different sub-phases of modelling is shown in Table 2. The three examples from Group 1 mentioned above are marked with bold numbers in the table.

**Table 2:** Concepts related to vertex-edge graphs and sub-phases of modelling in Group 1

Category	Transfer of the model	Metrics	Effectiveness: Doubles	Effectiveness: Omissions	Cover	Graph structure	Subgraph	Graph complexity	Duplicates	Start and end points	Directed graph	Passage sense	SUM
Understanding													0
Simplifying		2					1	**2**			1	1	7
Mathematising			**1**							**1**	1	1	4
Working mathematically			1		1			1			1		4
Interpreting	2		2		1			1					6
Validating		1	4	2				1		1			9
SUM	2	3	8	2	2	0	1	5	0	2	3	2	30

In Group 2, no concepts related to vertex-edge graphs were discernible in three sub-phases of modelling—namely in understanding, simplifying and interpreting (see Table 3). A conspicuously large number of references to vertex-edge graphs could be found in mathematical work and also in mathematising. Mathematical work was associated with all concepts related to vertex-edge graphs (except omissions and transferability). Particularly central to mathematical work were effectiveness and the start and end points. The discussions around start and end points would also have had a potential for further insights from a content perspective. An equally important sub-category was that of metrics, as three different sub-phases of modelling were also linked there. The above example from Group 2 is marked with a bold number in the table.

**Table 3:** Concepts related to vertex-edge graphs and sub-phases of modelling in Group 2

Category	Transfer of the model	Metrics	Effectiveness: Doubles	Effectiveness: Omissions	Cover	Subgraph	Graph complexity	Duplicates	Start and end points	Directed graph	Passage sense	SUM
Understanding												0
Simplifying												0
Mathematising		3			2	1		1	3			10
Working mathematically		4	**11**		1	1	3	5	12	1	1	39
Interpreting												0
Validating		3	2			1			3			9
SUM	0	10	13	0	3	3	3	6	18	1	1	58

Figures 7 and 8 illustrate the interaction of the modelling process and concepts with reference to vertex-edge graphs. Group-specifically, these were identified more in the mathematical work (Group 2) or in other sub-phases (Group 1). This is also illustrated by the values in Tables 2 and 3. Here, focal points from graph theory could be worked out, particularly in Group 2.

## Discussion

The analysis shows that concepts related to vertex-edge graphs were conspicuous and significant throughout the task processing of both groups. It can be seen that the most frequently identified concepts can be assigned to the area of edges of graphs. The question of the effectiveness of the chosen path plays a special role in the example chosen here, as the basic idea of optimisation was considered at the local level of the edges. Here the special chance of the treatment of discrete optimization problems in the school shows up (Schuster, 2004). It is a particular strength of the problem used that graph-theoretical concepts such as Euler path have a direct correspondence in the real problem (optimal path). Effectiveness can be seen here as a connecting element between mathematical and real models and, in this context, it establishes an interesting link between modelling and discrete problems. This shows the easy accessibility and lack of prerequisites for the processing (Gibson, 2012; van den Heuvel & Krabbendam, 1991), when an appropriate modelling problem is selected.

There are also differences between the groups, which can be seen, on the one hand, in the mathematical processing depth and, on the other, from the generalisability of the results obtained. For example, while Group 1 discussed the transfer of the model to other city districts or to other real situations in some places, Group 2 focused much more frequently on the structure of the graph. Substantial generalisable considerations were already made; however, these related only to arguments close to reality and not to the graph structure. Further generalisations at the level of the structure of the graph did not take place because not enough abstraction activities had been done. This shows the importance of modelling competences as a prerequisite for working with discrete concepts.

Overall, it should also be noted that the students were intensively concerned with the edges of the graph, although not with the nodes. This aspect can be of great importance from a mathematical perspective because it is precisely the structure of the nodes that is important in the classification of different vertex-edge graphs (for example, in the Euler path). On the one hand, it can be seen that this was a challenging problem that was at the same time easy to understand (Anderson et al., 2004), but on the other hand, its content was not fully exploited (low ‘floor’ … high ‘ceiling’-tasks; Ingram et al., 2020, p. 500). However, it must be considered that the students had no prior knowledge of this area of graph theory but developed these ideas and concepts only during the course of working on their task. It should be noted here that graph theory content is not part of the curriculum at German schools. Therefore, it is not surprising that students have no prior knowledge in this area (Kaiser et al., 2011). In contrast, the teaching of modelling competences is mandatory in Germany. This may explain why the students did not use more complex graph theoretic content (for example, Euler’s Theorem on Euler paths). The fact that they used these concepts at all well could be due to the easy accessibility (Gibson, 2012) or also to the intuitive approach, which, as observed in other areas, allows independent generalisations (Amit & Neria, 2008; Steele, 2008). However, it could be a particular strength of graph theory that one can intuitively understand central concepts.

This might have been supported by the existing modelling competences, as the work within modelling phases could mostly be identified at the same time as the usage of concepts related to graphs. This is a central point and shows an interdependence: discrete models can be better integrated if they are known, and they can be integrated only if the corresponding modelling competences are available. Only when both are present can the choice of discrete models in the context of the modelling processes discussed above succeed (see Sect. 2.3). The transcript excerpts presented above show, for example, that the discussion about roads that have to be covered twice has to be explored mathematically on one’s own first. With further knowledge about graph theory, the students could have directly accessed mathematical models and gone more deeply into the topic. The work could therefore have been more fruitful at both the content level and the process level. At the same time, it is remarkable that students without prior knowledge of graph theory and experience of working with it developed and used so many concepts related to vertex-edge graphs. This could also be seen in the intuitive approach of the students shown above, based on a drawing in the city map. This shows the specific potential of graph theory for mathematics teaching, which, in combination with open modelling problems, opens up access to a completely new mathematical subject area. Overall, this points to the mutual benefit of teaching modelling through discrete mathematics and the benefit of teaching discrete mathematics through modelling.

When looking at the sub-phases of the modelling cycles and concepts related to vertex-edge graphs, the differences between the groups become even more evident. It becomes clear that the references to vertex-edge graphs occurred less, for example, during the understanding phase than over the later course of the modelling process. There were also group-specific sub-phases in which references to vertex-edge graphs did not occur at all. By analysing the modelling phases and concepts of graphs jointly, specific students’ difficulties could be identified that could not be seen by examining the usage of vertex-edge graphs alone. For example, it was possible to determine the phase of the solution process at which the graph-theoretical aspects occurred or were missing. The analysis revealed the most important problematic aspects and topics students encountered. Based on these results, intervention methods for further modelling activities with graph-theoretical problems can be developed as scaffolding measures (Stender & Kaiser, 2015). Due to its process character, the modelling perspective offers the possibility for controlling the solution processes at the meta-level by using metacognitive aids (Beckschulte, 2020; Stillman, 2011; Vorhölter, Krüger, et al., 2019). For example, in the intuitive approach described above, a metacognitive aid to search for the mathematical model could open the view in the direction of more abstract generalisations. The modelling perspective thus offers the opportunity to describe the process character of developing answers to the original problems based on carrying out a modelling cycle (Kaiser & Stender, 2013) and its sub-processes, in addition to looking at the content of the students’ solution processes. This allows feedback to be provided to the students or focus to be placed on metacognitive strategies (Vorhölter, Krüger, et al., 2019).

The study presented here is qualitatively orientated, based on a single case-study, and thus it has limited potential for more general results. Only a few students were observed, and only one modelling example was used. Due to the case-study nature of the study, quantitatively derived results were not the aim, which was rather an analysis based on the central question in which sub-phases of modelling and concepts related to vertex-edge graphs were used by the students, especially simultaneously. Further investigations with other modelling examples—also from other areas of discrete mathematics—and more students will be necessary to expand the scope of the study. It would also be interesting to use examples of modelling problems related to vertex-edge graphs with digital technology (Durcheva & Varbanova, 2017; Geschke et al., 2005).

## Conclusion

Graph-theoretical modelling problems like the example used here are not part of the content prescribed in the German curriculum for mathematics education. However, they do offer the potential to stimulate substantial mathematical reflections and also to motivate students with the help of real-world experiences. Such problems are therefore very interesting as content for mathematics education and offer the opportunity to introduce students to optimization problems already early in their school career. At the same time, the study showed that intensive and different modelling processes were stimulated and carried out. So, from a modelling perspective, examples like the one used in this study are of high educational value. In addition, the students were motivated and mathematically active during the long processing periods of several hours over the two days. This also shows the potential to promote high motivation through suitable modelling problems (Ferrarello & Mammana, 2018). Thus, the advantages of discrete mathematics for mathematics teaching may be exemplified across three levels (Hart & Martin, 2018), namely, interesting content (the example from graph theory), the development of process-related competences (concerning modelling competences) and the level of affect-related goals (motivation).

Further studies with larger sample numbers and integration into design projects (Cobb et al., 2003) are needed to show more clearly the benefits of modelling examples using discrete mathematics, and also to attract teachers more strongly to discrete mathematics in general and graph theory in particular (Gaio & Di Paola, 2018). A quantitative study with more students and a variety of examples could also be useful here.

The examples of discrete mathematics cannot yet be fully used by students—also in connection with modelling processes—because of missing prior knowledge of graph theory. Here, prior knowledge could enable the use of more advanced mathematical models and thus further mathematical and extra-mathematical discoveries. The process character of mathematical modelling can support work with discrete concepts. Mathematical modelling offers a chance to take a new look at discrete mathematics content for schools and present it as a central example of successful modelling processes, not least because modelling competences ultimately represent a central component in many educational standards all over the world (KMK, 2012; Lu & Kaiser, 2021; NCTM, 2000).

## References

[CR1] Amit, M., & Neria, D. (2008). “Rising to the challenge”: Using generalization in pattern problems to unearth the algebraic skills of talented pre-algebra students. *ZDM—Mathematics Education*, *40*(1), 111–129. 10.1007/s11858-007-0069-5

[CR2] Anderson, I., van Asch, B., & van Lint, J. (2004). Discrete mathematics in the high school curriculum. *ZDM—Mathematics Education*, *36*(3), 105–116. 10.1007/BF02652778

[CR3] Beckschulte, C. (2020). Mathematical modelling with a solution plan: An intervention study about the development of grade 9 students’ modelling competencies. In G. Stillman, G. Kaiser, & C. E. Lampen (Eds.), *Mathematical modelling education and sense-making* (pp. 129–138). Springer International Publishing. 10.1007/978-3-030-37673-4_12

[CR4] Bergman Ärlebäck, J., & Bergsten, C. (2010). On the use of realistic Fermi problems in introducing mathematical modelling in upper secondary mathematics. In R. Lesh, P. L. Galbraith, C. R. Haines, & A. Hurford (Eds.), *Modeling students’ mathematical modeling competencies* (pp. 597–609). Springer. 10.1007/978-1-4419-0561-1_52

[CR5] Beutelspacher A, Zschiegner M-A (2014). Diskrete Mathematik für Einsteiger. Springer.

[CR6] Caron, F. (2019). Approaches to investigating complex dynamical systems. In G. Stillman & J. P. Brown (Eds.), *Lines of inquiry in mathematical modelling research in education* (pp. 83–103). Springer. 10.1007/978-3-030-14931-4_5

[CR7] Castillo-Garsow C, Johnson HL, Moore KC (2013). Chunky and smooth images of change. For the Learning of Mathematics.

[CR8] Cevikbas M, Kaiser G, Schukajlow S (2021). A systematic literature review of the current discussion on mathematical modelling competencies: state-of-the-art developments in conceptualizing, measuring, and fostering. Educational Studies in Mathematics.

[CR9] Chick HL, Watson JM (2003). Stochastics education: growth, goals, and gaps in a maturing discipline. Mathematics Education Research Journal.

[CR10] Cobb P, Confrey J, diSessa A, Lehrer R, Schauble L (2003). Design experiments in educational research. Educational Researcher.

[CR11] Coenen, T., Hof, F., & Verhoef, N. (2018). Combinatorial reasoning to solve problems. In E. W. Hart & J. Sandefur (Eds.), *Teaching and learning discrete mathematics worldwide: Curriculum and research* (pp. 69–79). Springer. 10.1007/978-3-319-70308-4_5

[CR12] DeBellis, V. A., & Rosenstein, J. G. (2004). Discrete mathematics in primary and secondary schools in the United States. *ZDM—Mathematics Education*, *36*(2), 46–55. 10.1007/BF02655758

[CR13] Dolgos KA (1990). Discrete mathematics in the high school curriculum. International Journal of Mathematical Education in Science and Technology.

[CR14] Doorman, L. M., & Gravemeijer, K. P. E. (2009). Emergent modeling: discrete graphs to support the understanding of change and velocity. *ZDM—Mathematics Education*, *41*(1), 199–211. 10.1007/s11858-008-0130-z

[CR15] Dresing, T., & Pehl, T. (2018). *Praxisbuch Interview, Transkription & Analyse: Anleitungen und Regelsysteme für qualitativ Forschende* (8. Auflage). www.audiotranskription.de/praxisbuch

[CR16] Durcheva M, Varbanova E (2017). Applications of CAS in the teaching and learning of discrete mathematics. Mathematics in Computer Science.

[CR17] Ferrarello, D., & Mammana, M. F. (2018). Graph theory in primary, middle, and high school. In E. W. Hart & J. Sandefur (Eds.), *Teaching and learning discrete mathematics worldwide: Curriculum and research* (pp. 183–200). Springer. 10.1007/978-3-319-70308-4_12

[CR18] Gaio, A., & Di Paola, B. (2018). Discrete mathematics in lower school grades? Situation and possibilities in italy. In E. W. Hart & J. Sandefur (Eds.), *Teaching and learning discrete mathematics worldwide: Curriculum and research* (pp. 41–51). Springer. 10.1007/978-3-319-70308-4_3

[CR19] Galbraith, P. L., & Stillman, G. (2006). A framework for identifying student blockages during transitions in the modelling process. *ZDM—Mathematics Education*, *38*(2), 143–162. 10.1007/BF02655886

[CR20] Geschke, A., Kortenkamp, U., Lutz-Westphal, B., & Materlik, D. (2005). Visage—Visualization of algorithms in discrete mathematics. *ZDM—Mathematics Education*, *37*(5), 395–401. 10.1007/s11858-005-0027-z

[CR21] Gibson, J. P. (2012). Teaching graph algorithms to children of all ages. *Annual Conference on Innovation and Technology in Computer Science Education, ITiCSE*, 34–39. 10.1145/2325296.2325308

[CR22] Goldin, G. A. (2004). Problem solving heuristics, affect, and discrete mathematics. *ZDM—Mathematics Education*, *36*(2), 56–60. 10.1007/BF02655759

[CR23] Greefrath, G., Hertleif, C., & Siller, H.-S. (2018). Mathematical modelling with digital tools—A quantitative study on mathematising with dynamic geometry software. *ZDM—Mathematics Education*, *50*(1–2), 233–244. 10.1007/s11858-018-0924-6

[CR24] Greefrath, G., Kaiser, G., Blum, W., & Borromeo Ferri, R. (2013). Mathematisches Modellieren—Eine Einführung in theoretische und didaktische Hintergründe. In R. Borromeo Ferri, G. Greefrath, & G. Kaiser (Eds.), *Mathematisches Modellieren für Schule und Hochschule* (pp. 11–37). Springer. 10.1007/978-3-658-01580-0_1

[CR25] Greefrath, G., & Siller, H.-S. (2017). Modelling and simulation with the help of digital tools. In G. Stillman, W. Blum, & G. Kaiser (Eds.), *Mathematical modelling and applications* (pp. 529–539). Springer. 10.1007/978-3-319-62968-1_44

[CR26] Greubel, A., Siller, H.-S., & Hennecke, M. (2020). Teaching simulation literacy with evacuations: Concept, technology, and material for a novel approach. In C. Alario-Hoyos, M. J. Rodríguez-Triana, M. Scheffel, I. Arnedillo-Sánchez, & S. M. Dennerlein (Eds.), *Addressing global challenges and quality education* (pp. 200–214). Springer. 10.1007/978-3-030-57717-9_15

[CR27] Hankeln C, Greefrath G (2021). Mathematische Modellierungskompetenz fördern durch Lösungsplan oder Dynamische Geometrie-Software? Empirische Ergebnisse aus dem LIMo-Projekt. Journal für Mathematik-Didaktik.

[CR28] Hart, E. W., & Martin, W. G. (2018). Discrete mathematics is essential mathematics in a 21st century school curriculum. In E. W. Hart & J. Sandefur (Eds.), *Teaching and learning discrete mathematics worldwide: Curriculum and research* (pp. 3–19). Springer. 10.1007/978-3-319-70308-4_1

[CR29] Ingram N, Holmes M, Linsell C, Livy S, McCormick M, Sullivan P (2020). Exploring an innovative approach to teaching mathematics through the use of challenging tasks: A New Zealand perspective. Mathematics Education Research Journal.

[CR30] James DJG, Wilson MA, Berry JS, Burghes DN, Huntley ID, James DJG, Moscardini AO (1986). Continuous and discrete techniques in mathematical modelling. Mathematical modelling methodology, models and micros.

[CR31] Kaiser, G. (2007). Modelling and modelling competencies in school. In C. R. Haines, P. L. Galbraith, W. Blum, & S. Khan (Eds.), *Mathematical modelling (ICTMA 12): Education, engineering and economics* (pp. 110–119). Horwood. 10.1533/9780857099419.3.110

[CR32] Kaiser G, Cai J (2017). The teaching and learning of mathematical modeling. Compendium for research in mathematics education.

[CR33] Kaiser, G. (2020). Mathematical modelling and applications in education. In S. Lerman (Ed.), *Encyclopedia of mathematics education* (pp. 553–561). Springer. 10.1007/978-3-030-15789-0_101

[CR34] Kaiser, G., Schwarz, B., & Buchholtz, N. (2011). Authentic modelling problems in mathematics education. In G. Kaiser, W. Blum, R. Borromeo Ferri, & G. Stillman (Eds.), *Trends in teaching and learning of mathematical modelling* (Vol. 1, pp. 591–601). Springer. 10.1007/978-94-007-0910-2_57

[CR35] Kaiser, G., & Stender, P. (2013). Complex modelling problems in co-operative, self-directed learning environments. In G. Stillman, G. Kaiser, W. Blum, & J. P. Brown (Eds.), *Teaching mathematical modelling: Connecting to research and oractice* (pp. 277–293). Springer. 10.1007/978-94-007-6540-5_23

[CR36] Keune, M., & Henning, H. (2003). Modelling and spreadsheet calculation. In Q.-X. Ye, W. Blum, K. Houston, & Q.-Y. Jiang (Eds.), *Mathematical Modelling in Education and Culture: ICTMA 10* (pp. 101–110). Horwood. 10.1533/9780857099556.3.99

[CR37] KMK (Ed.). (2012). *Bildungsstandards im Fach Mathematik für die Allgemeine Hochschulreife (Beschluss der Kultusministerkonferenz vom 18.10.2012).* Wolters Kluwer.

[CR38] Kuckartz, U. (2014). *Qualitative text analysis: A guide to methods, practice & using software*. SAGE.

[CR39] Lu X, Kaiser G (2021). Creativity in students’ modelling competencies: Conceptualisation and measurement. Educational Studies in Mathematics.

[CR40] Maaß, K. (2006). What are modelling competencies? *ZDM—Mathematics Education*, *38*(2), 113–142. 10.1007/BF02655885

[CR41] Medová J, Páleníková K, Rybanský L, Naštická Z (2019). Undergraduate students’ solutions of modeling problems in algorithmic graph theory. Mathematics.

[CR42] NCTM (Ed.). (2000). *Principles and standards for school mathematics*. National Council of Teachers of Mathematics.

[CR43] Niss, M. (2013). Modeling a crucial aspect of students’ mathematical modeling. In R. Lesh, P. L. Galbraith, C. R. Haines, & A. Hurford (Eds.), *Modeling students’ mathematical modeling competencies* (pp. 43–59). Springer. 10.1007/978-94-007-6271-8_4

[CR44] Niss, M., Blum, W., & Galbraith, P. L. (2007). Introduction. In W. Blum, P. L. Galbraith, H.-W. Henn, & M. Niss (Eds.), *Modelling and applications in mathematics education. The 14th ICMI Study* (Vol. 10, pp. 3–32). Springer. 10.1007/978-0-387-29822-1_1

[CR45] Ortlieb CP (2004). Mathematische Modelle Und Naturerkenntnis. Mathematica Didactica.

[CR46] Ouvrier-Buffet, C. (2020). Discrete mathematics teaching and learning. In S. Lerman (Ed.), *Encyclopedia of mathematics education* (pp. 227–233). Springer. 10.1007/978-3-030-15789-0_51

[CR47] Pohjolainen, S., & Heiliö, M. (2016). Introduction. In S. Pohjolainen (Ed.), *Mathematical modelling* (pp. 1–5). Springer. 10.1007/978-3-319-27836-0_1

[CR48] Pollak, H. O. (1977). The interaction between mathematics and other school subjects (including integrated courses). In H. Athen & H. Kunle (Eds.), *Proceedings of the Third International Congress on Mathematical Education* (pp. 255–264). Zentralblatt für Didaktik der Mathematik.

[CR49] Pollak, H. O. (2007). Mathematical modelling—A conversation with Henry Pollak. In W. Blum, P. L. Galbraith, H.-W. Henn, & M. Niss (Eds.), *Modelling and applications in mathematics education* (pp. 109–120). Springer. 10.1007/978-0-387-29822-1_9

[CR50] Ruzika, S., Siller, H.-S., & Bracke, M. (2017). Evakuierungsszenarien in Modellierungswochen—ein interessantes und spannendes Thema für den Mathematikunterricht. In H. Humenberger & M. Bracke (Eds.), *Neue Materialien für einen realitätsbezogenen Mathematikunterricht 3* (pp. 181–190). Springer. 10.1007/978-3-658-11902-7_14

[CR51] Schuster, A. (2004). About traveling salesmen and telephone networks—Combinatorial optimization problems at high school. *ZDM—Mathematics Education*, *36*(2), 77–81. 10.1007/BF02655762

[CR52] Sinclair, N., & Jackiw, N. (2010). Modeling practices with the geometer’s sketchpad. In R. Lesh, P. L. Galbraith, C. R. Haines, & A. Hurford (Eds.), *Modeling students’ mathematical modeling competencies* (pp. 541–554). Springer. 10.1007/978-1-4419-0561-1_47

[CR53] Steele, D. (2008). Seventh-grade students’ representations for pictorial growth and change problems. *ZDM – Mathematics Education*, *40*(1), 97–110. 10.1007/s11858-007-0063-y

[CR54] Stender, P., & Kaiser, G. (2015). Scaffolding in complex modelling situations. *ZDM—Mathematics Education*, *47*(7), 1255–1267. 10.1007/s11858-015-0741-0

[CR55] Stillman, G. (2011). Applying metacognitive knowledge and strategies in applications and modelling tasks at secondary school. In G. Kaiser, W. Blum, R. Borromeo Ferri, & G. Stillman (Eds.), *Trends in teaching and learning of mathematical modelling* (Vol. 1, pp. 165–180). Springer. 10.1007/978-94-007-0910-2_18

[CR56] Street, A. P., & Street, D. J. (1998). Discrete approaches to mathematical modelling. In P. L. Galbraith, W. Blum, G. Booker, & I. D. Huntley (Eds.), *Mathematical modelling. Teaching and assessment in a technology-rich world* (pp. 207–219). Horwood.

[CR57] Thomas, M. O. J., de Freitas Druck, I., Huillet, D., Ju, M.-K., Nardi, E., Rasmussen, C., & Xie, J. (2015). Key mathematical concepts in the transition from secondary school to university. In S. J. Cho (Ed.), *The proceedings of the 12th international congress on mathematical education* (pp. 265–284). Springer International Publishing. 10.1007/978-3-319-12688-3_18

[CR58] Van den Heuvel G, Krabbendam H, Niss M, Blum W, Huntley I (1991). Introducing discrete graphs to 12 year olds. Teaching of mathematical modelling and applications.

[CR59] Vorhölter, K., Greefrath, G., Borromeo Ferri, R., Leiß, D., & Schukajlow, S. (2019a). Mathematical modelling. In H. N. Jahnke & L. Hefendehl-Hebeker (Eds.), *Traditions in German-speaking mathematics education research* (pp. 91–114). Springer. 10.1007/978-3-030-11069-7_4

[CR60] Vorhölter, K., Krüger, A., & Wendt, L. (2019b). Chapter 2: Metacognition in mathematical modeling—An overview. In S. A. Chamberlin & B. Sriraman (Eds.), *Affect in mathematical modeling* (pp. 29–51). Springer. 10.1007/978-3-030-04432-9_3

[CR61] Weigand, H.-G. (2004). Sequences—Basic elements for discrete mathematics. *ZDM—Mathematics Education*, *36*(3), 91–97. 10.1007/BF02652776

[CR62] Weigand, H.-G. (2014). A discrete approach to the concept of derivative. *ZDM—Mathematics Education*, *46*(4), 603–619. 10.1007/s11858-014-0595-x

[CR63] Ziegenbalg J, Berry JS, Burghes DN, Huntley ID, James DJG, Moscardini AO (1984). Discrete modelling, difference equations and the use of computers in mathematical education. Teaching and applying mathematical modelling.

